# Should we give antibiotics to neonates with mild non-progressive symptoms? A comparison of serial clinical observation and the neonatal sepsis risk calculator

**DOI:** 10.3389/fped.2022.882416

**Published:** 2022-07-22

**Authors:** Alberto Berardi, Isotta Zinani, Luca Bedetti, Eleonora Vaccina, Alessandra Toschi, Greta Toni, Marco Lecis, Federica Leone, Francesca Monari, Michela Cozzolino, Tommaso Zini, Alessandra Boncompagni, Lorenzo Iughetti, Francesca Miselli, Licia Lugli

**Affiliations:** ^1^Neonatal Intensive Care Unit, Women's and Children's Health Department, Azienda Ospedaliero-Universitaria Policlinico, Modena, Italy; ^2^Pediatric Post-Graduate School, Università degli Studi di Modena e Reggio Emilia, Modena, Italy; ^3^Doctorate School, Clinical and Experimental Medicine, University of Modena and Reggio Emilia, Modena, Italy; ^4^Obstetrics Unit, Mother Infant Department, University Hospital Policlinico of Modena, Modena, Italy; ^5^Pediatric Unit, Women's and Children's Health Department, Azienda Ospedaliero-Universitaria Policlinico, Modena, Italy

**Keywords:** early-onset sepsis, neonatal early-onset sepsis calculator, newborn, serial clinical observation, neonates, perinatal distress

## Abstract

**Objective:**

To compare two strategies [the neonatal sepsis risk calculator (NSC) and the updated serial clinical observation approach (SCO)] for the management of asymptomatic neonates at risk of early-onset sepsis (EOS) and neonates with mild non-progressive symptoms in the first hours of life.

**Methods:**

This was a single-center, retrospective cohort study conducted over 15 months (01/01/2019–31/03/2020). All live births at ≥34 weeks of gestation were included. Infants were managed using SCO and decisions were compared with those retrospectively projected by the NSC. The proportion of infants recommended for antibiotics or laboratory testing was compared in both strategies. McNemar's non-parametric test was used to assess significant differences in matched proportions.

**Results:**

Among the 3,445 neonates (late-preterm, *n* = 178; full-term, *n* = 3,267) 262 (7.6%) presented with symptoms of suspected EOS. There were no cases of culture-proven EOS. Only 1.9% of the neonates were treated with antibiotics (median antibiotic treatment, 2 days) and 4.0% were evaluated. According to NSC, antibiotics would have been administered in 5.4% of infants (absolute difference between SCO and NSC, 3.51%; 95% CI, 3.14–3.71%; *p* <0.0001) and 5.6% of infants would have undergone “rule out sepsis” (absolute difference between SCO and NSC, 1.63%, 95% CI 1.10–2.05; *p* <0.0001).

**Conclusion:**

SCO minimizes laboratory testing and unnecessary antibiotics in infants at risk of EOS or with mild non-progressive symptoms, without the risk of a worse neonatal outcome. The NSC recommends almost three times more antibiotics than the SCO without improving neonatal outcomes.

## Introduction

The current management of neonates at risk of early-onset sepsis (EOS) remains controversial, and the suggested approaches are heterogeneous (1). EOS rates have declined substantially due to the widespread use of intrapartum antibiotic prophylaxis (IAP). However, the diagnosis of EOS remains challenging because its initial clinical signs may be ambiguous, diagnostic tests are poorly predictive, and delayed antibiotic treatment can have devastating consequences. Therefore, 30–40 uninfected neonates are exposed to unnecessary antibiotics for each infant subsequently confirmed to have EOS (2). However, perinatal antibiotic exposure is a major concern, given the potential long-term effects of changes in the intestinal flora of uninfected neonates (1, 3) and the consensus on the optimal management of neonates considered at risk of EOS is shifting (4, 5).

The American Academy of Pediatrics suggests three alternative approaches for the use of risk factors (RFs) to identify infants at increased risk of EOS (1). First, the categorical RFs assessment (6); such an approach has some limitations and is associated with higher rates of antibiotic treatment (7). Second, the neonatal sepsis risk calculator (NSC). The NSC has a Bayesian approach to create a multivariate model to predict infant-specific EOS risk, derived and validated from a case-control study of blood culture-proven EOS. NSC permit improved delineation of low-risk babies that can be safely managed with observation. Following the adoption of the most recent NSC laboratory workups, the use of empirical antibiotic administration in the first 24 h of life has decreased significantly (from 5.0 to 2.6%) in a recent US multicenter study (8). However, these low rates of antibiotic treatment have rarely been confirmed outside the USA (the country where NSC was created), and antibiotic treatment may reach 8% of neonates elsewhere (9). Finally, increasing evidence has shown that asymptomatic neonates at risk can be safely managed with serial clinical observation (SCO) without antibiotic treatment (10–12). However, for “less symptomatic” infants, it is difficult to allow adequate time to undergo a physiological transition before deciding whether clinical signs are transient or permanent (4).

This study aimed to assess the impact of an updated SCO approach planned to reduce unnecessary antibiotic therapy in “less symptomatic” full-term and late-preterm neonates. The impact on neonatal outcomes, laboratory testing rates, and neonatal intensive care unit (NICU) admissions were also evaluated. Furthermore, we compared management decisions using the updated SCO with those projected through the virtual application of the NSC.

## Methods

### Study design

This was a retrospective study carried out over 15 months (from January 1^st^, 2019 to March 31^th^, 2020), in a single, high-volume tertiary care center (Modena University Hospital, Italy), with ~3,000 live births/year. This center advocates a recto-vaginal culture screening strategy at 35–37 weeks gestation (6). The project was approved by the local ethics committee (no. 169/2019/OSS^*^/AOUMO). All infants born at ≥34 weeks gestation were included in the study. To obtain complete information on the rates of antibiotic treatment in the entire population, we included neonates with malformations, metabolic diseases, or surgical complications. Full maternal data (gestational age, mode of delivery, group B Streptococcus status, RFs for EOS, and duration of IAP) were routinely recorded in neonatal charts. The records were collected anonymously in an Excel format with controlled access, assigning each newborn a progressive numerical code.

### SCO approach

This approach is directed at asymptomatic infants with RFs (10). A standardized form, signed by each examiner, were used to detail general wellbeing, skin color, and respiratory signs at standard intervals (at ages 1, 3, 6, 12, 18, 24, 36, and 48 h) (13). All asymptomatic neonates with RFs for EOS are usually managed by midwives, nurses or pediatricians in the mother baby unit, where neonates ≥ 35 weeks' gestation “room in” with their own mother. Asymptomatic neonates with 34 weeks' gestation are usually admitted to intermediate care unit.

Each newborn with symptoms of suspected sepsis is immediately referred to a neonatal care specialist. However, those with mild to moderate disease that requires oxygen support or a high-flow nasal cannula are separated from their mothers and admitted to an intermediate care unit. Severely ill neonates and those undergoing nCPAP or mechanical ventilation are admitted to the NICU. Ampicillin plus an aminoglycoside is administered as empiric therapy for suspected EOS in symptomatic neonates. Before March 2018, white blood cell count (WBC), C-reactive protein (CRP) levels, and blood culture were obtained to rule out sepsis in asymptomatic, chorioamnionitis-exposed neonates or those who developed symptoms of variable severity.

### Updated SCO approach

In March 2018, the main neonatal symptoms triggering laboratory evaluation were defined (Table 1A) (14). Neonates with mild, non-progressive symptoms (that can be due to non-infectious diseases, i.e., transient tachypnea of the newborn) who present in the first few hours of life can be reevaluated at 2-h intervals. No laboratory testing is performed, and no antibiotic therapy is given if symptoms remain mild, even after multiple re-evaluations in case of minor criteria. In contrast, the presence of major symptoms (as defined in Table 1A) or worsening of mild symptoms suggest the need for laboratory evaluation. Among patients who undergo a sepsis workup, treatment decisions are left to the discretion of the physician. This updated approach aims to minimize laboratory testing (which, in our experience, strongly influences clinicians' decisions) (10), unnecessary antibiotics, and mother-baby separation.

**Table 1:** Symptoms and classification of infant's clinical presentation according to serial clinical observation and neonatal sepsis calculator.

**(A)** Minor and major clinical symptoms and criteria suggesting observation or laboratory evaluation and antibiotic treatment [modified from Berardi et al. (14)].	
**Minor** ^*^	**Major**
Mild respiratory distress (> 60/m) without the need of respiratory support	Moderate to severe respiratory distress (requiring respiratory support)^§^ → tachypnoea plus increased respiratory effort
Tachycardia > 160 bpm	Hypoxia, reduced SpO2 saturation
Metabolic acidosis (base excess ≤ −10 mmol/l)	Reduced skin perfusion, Refill time ≥ 3 seconds, Signs of shock
Temperature <36° or > 37.5 <38 °C	Temperature ≥ 38 °C
	Grayish, pallor or marbling of the skin color
	Worsening of general wellbeing, apnoea, lethargy, irritability, convulsions
**(B)** Classification of infant's clinical presentation according to NSC (available at https://neonatalsepsiscalculator.kaiserpermanente.org).	
**Clinical exam**	**Description**
Clinical illness	1. Persistent need for NCPAP / HFNC / mechanical ventilation (outside of the delivery room)
	2. Hemodynamic instability requiring vasoactive drugs
	3. Neonatal encephalopathy /Perinatal depression: - Seizure - Apgar Score at 5 min <5
	4. Need for supplemental O2 > 2 h to maintain oxygen saturations > 90% (outside of the delivery room)
Equivocal	1. Persistent physiologic abnormality > 4 h - Tachycardia (HR > 160) - Tachypnea (RR > 60) - Temperature instability (> 100.4°F or <97.5 °F) - Respiratory distress (grunting, flaring, or retracting) not requiring supplemental O2
	2. Two or more physiologic abnormalities lasting for > 2 h Tachycardia (HR > 160) - Tachypnea (RR > 60) - Temperature instability (> 100.4°F or <97.5 °F) - Respiratory distress (grunting, flaring, or retracting) not requiring supplemental O2
	Note: abnormality can be intermittent
Well appearing	No persistent physiologic abnormalities

*SpO_2_, Saturation of peripheral oxygen*.

* *On the basis of the clinician's judgment, laboratory evaluation can be delayed in the presence of minor, initial, unspecific and non-progressive symptoms during the first 12–24 h of life. Neonates with mild symptoms are re-evaluated at 2-h intervals. The presence of major symptoms and the worsening or persistence (for 12¬24 h) of minor symptoms warrant laboratory evaluation and (eventually) empirical antibiotics, but the decision is left to the clinician's discretion*.

§ *Respiratory support includes mechanical ventilation. However, it does not necessarily include high flow nasal cannula or nasal continuous positive airway pressure*.

*HFNC, High Flow Nasal Cannula; HR, Heart Rate; NCPAP, Nasal Continuous Positive Airway Pressure; RR, Respiratory Rate*.

### NSC approach

The NSC is an online tool that quantifies the risk of EOS in infants with a gestational age ≥34 weeks using a pretest probability. Recommendations for antibiotic treatment or neonatal management are derived from an algorithmic framework based on the local incidence of EOS, maternal RFs (gestational age, highest intrapartum temperature, duration of membrane rupture, GBS status, and IAP), and clinical presentation of the infants during the first few hours of life. For each infant, the previous risk of EOS was calculated based on the local incidence of EOS and maternal RFs alone. The prior probability is converted into a posterior probability of EOS in the different categories of infants' clinical presentation (likelihood ratio of 0.41, 5.0, and 21.2 for well-appearing, equivocal, and clinical illness, respectively; Table 1B) (15). The resulting post-test probability of EOS is classified into three risk layers (<0.65, 0.65–1.54, and >1.54 cases/1,000 live births). The management recommendations suggested by the NSC are as follows: (1) no culture, no antibiotics, routine vitals (posterior risk <1/1,000 live births); (2) no culture, no antibiotics, vitals every 4 h for 24 h (posterior risk <1/1,000 live births, but prior risk >1/1,000 live births); (3) blood culture, vitals every 4 h for 24 h (posterior risk 1–3/1,000 live births); (4) strongly consider starting empiric antibiotics, vitals per NICU (posterior risk <3/1,000 live births); and (5) empiric antibiotics, vitals per NICU (posterior risk >3/1,000 live births or clinical signs of illness). In the current study, each infant was retrospectively scored as well as appeared equivocal or clinically ill within 4 h after birth. Recommendations for the management of neonates according to NSC were calculated by assuming an incidence rate of EOS of 0.6/1,000 live births (16).

### Statistical analyses

We used MedCalc version 9.3 (MedCalc Software, https://www.medcalc.org). Continuous variables are expressed as mean ± SD or median and interquartile range (IQR), and categorical data are expressed as numbers (percentages). Categorical and continuous variables were compared between patient groups using the χ^2^ test, Fisher's exact test, Student's *t*-test, or Mann–Whitney test, as appropriate. All *p*-values refer to two-tailed tests of significance; *p* <0.05 was considered significant. McNemar's non-parametric test was used to assess significant differences in the matched proportions.

## Results

### All neonates

During the study period, 3,456 neonates were ≥34 weeks of gestation. Records were available for 3,445 (99.7%) infants, of which 178 were born late preterm and 3,267 were born full-term. The median gestational age was 39.6 weeks and the median birth weight was 3,310 g.

Table 2 shows demographics according to full-term or late-preterm delivery. Vaginal delivery and prenatal vagino-rectal screening were more likely in full-term neonates, while prolonged membrane rupture and IAP were more likely among late preterm neonates. Among the 3,445 infants included in the study, 264 (7.6%) had symptoms of suspected EOS (most were respiratory and already at birth). Table 3 shows the age of presentation of symptoms, NSC scores, and antibiotics administered by comparing full-term and late-preterm neonates. Only 1.9% of the entire cohort was treated with antibiotics (median 2 days), and 4% underwent “rule out sepsis”; 3.1 and 2.3% were admitted to the NICU or intermediate care unit, respectively (neonates who were admitted and were given antibiotics or underwent “rule out sepsis” are reported in the footnote of Table 3).

**Table 2:** Demographics, risk factors for EOS and intrapartum antibiotic prophylaxis.

	**Late preterm neonates (*n* = 178)**	**Full term neonates (*n* = 3,267)**	***p***	**All (*n* = 3,445)**
Median gestational age, wks	36.14 (35.29–36.57)	39.71 (39.0–40.29)	NA	39.57 (38.86–40.29)
Median birth weight, g	2,582.5 (2,295–2,860)	3,340 (3,060–3,620)	NA	3,310 (3,010–3,605)
Vaginal delivery	96 (53.93)	2,565 (78.49)	<0.0001	2,661 (77.24)
Prenatal vagino-rectal screening	66 (37.07)	3213 (98.35)	<0.0001	3,279 (95.17)
GBS positive screening	13 (19.69)	703 (21.88)	0.7838	716 (21.84)
GBS bacteriuria	0 (0)	46 (1.41)	0.2082	46 (1.34)
Prolonged membrane rupture	41 (23.03)	473 (15.31)	0.0044	514 (15.73)
Maternal temperature ≥ 38° C	2 (0.01)	34 (1.05)	0.9186	36 (1.05)
Previous infant with GBS disease	0 (0)	2 (0.06)	0.2050	2 (0.05)
At least 1 risk factor	178 (100)	503 (15.39)	NA	681 (19.77)
IAP	98 (55.05)	923 (28.25)	<0.0001	1,021 (29.63)
Adequate IAP	59 (60.20)	516 (55.90)	0.4785	575 (56.32)

*GBS, group B streptococcus; IAP, Intrapartum Antibiotic Prophylaxis; NA, not assessable; wks, weeks*.

*Highest maternal temperature and duration of membrane rupture were missing for 28 (0.8%) (1 preterm, 27 full term) and 178 (5.2%, all full term) cases, respectively. Percentages are calculated without missing cases*.

*Data are presented as median (IQR) and n (%)*.

**Table 3:** Age at presentation of symptoms, NSC score and antibiotics.

	**Late preterm neonates** **(*****n*** = **178)**	**Full term neonates** **(*****n*** = **3,267)**	**p**	**All** **(*****n*** = **3,445)**
Neonates with symptoms	53 (29.78)	211 (6.46)	<0.0001	264 (7.66)
Neonates with symptoms already at birth	46 (86.79)	134 (63.51)		180 (68.18)
Neonates with symptoms from 1 to 6 h of life	5 (9.43)	44 (20.85)	0.0044	49 (18.56)
Neonates with symptoms after 6 h of life	2 (3.77)	33 (15.64)		35 (13.26)
Serial clinical observation	56 (31.46)	301 (9.21)	<0.0001	357 (10.4)
Evaluation to rule out sepsis ^†^	33 (18.54)	104 (3.18)	<0.0001	137 (3.98)
NSC score <0.5	117 (70.06)	3,146 (96.30)	<0.0001	3,263 (94.72)
NSC score > 0.51 <1	11 (6.59)	46 (1.41)	<0.0001	57 (1.65)
NSC score > 1.01 <3	16 (9.58)	46 (1.41)	<0.0001	62 (1.80)
NSC score > 3.01	23 (13.77)	29 (0.89)	<0.0001	52 (1.51)
Neonates given antibiotics ^¶^	15 (8.43)	49 (1.49)	<0.0001	64 (1.86)
Median days on antibiotics	2 (2–2)	3 (2–3)	0.0637	2 (2–3)
Symptomatic neonates admitted to NICU ^§^	35 (19.66)	73 (2.23)	<0.0001	108 (3.13)
Symptomatic neonates admitted to intermediate care unit ^§^	17 (9.55%)	62 (1.90%)	<0.0001	79 (2.30%)
Symptomatic neonates unadmitted to NICU or intermediate care unit ^¥^	1 (0.06 %)	76 (2.33%)	0.1209	77 (2.24%)

*NICU, neonatal intensive care unit; NSC, neonatal sepsis calculator*.

† * Including white blood cell count, blood culture, and C-reactive protein*.

¶ * Among 64 neonates receiving antibiotics, 14 underwent therapeutic hypothermia and 6 had surgical prophylaxis*.

§ * Antibiotics were given to 55 out of 108 symptomatic neonates admitted to NICU and 9 out of 79 admitted to intermediate care unit; “rule out sepsis” was performed in all symptomatic neonates admitted to NICU*.

¥ * “Rooming in” was allowed within a few hours of birth to all 77 neonates and they were discharged home with a “healthy newborn” code*.

*Data are presented as median (IQR) or n (%)*.

Among the 200 symptomatic neonates unexposed to antibiotics, 77 (38%) were allowed to “room in” with their mothers within a few hours after birth. The remaining 123 (62%) were admitted to NICU (of which 80 underwent nCPAP) and/or intermediate care unit (of which 67 underwent high-flow nasal cannulation). Symptoms improved substantially within the first 24–48 h of life (median duration 72 h, IQR 24–120). Late-preterm neonates were more likely to have symptoms, undergo SCO, be evaluated, have a higher NSC score, be admitted to the NICU, and be treated with antibiotics. However, the median number of days on antibiotics did not differ between late preterm and full-term neonates.

**Figure 1:**
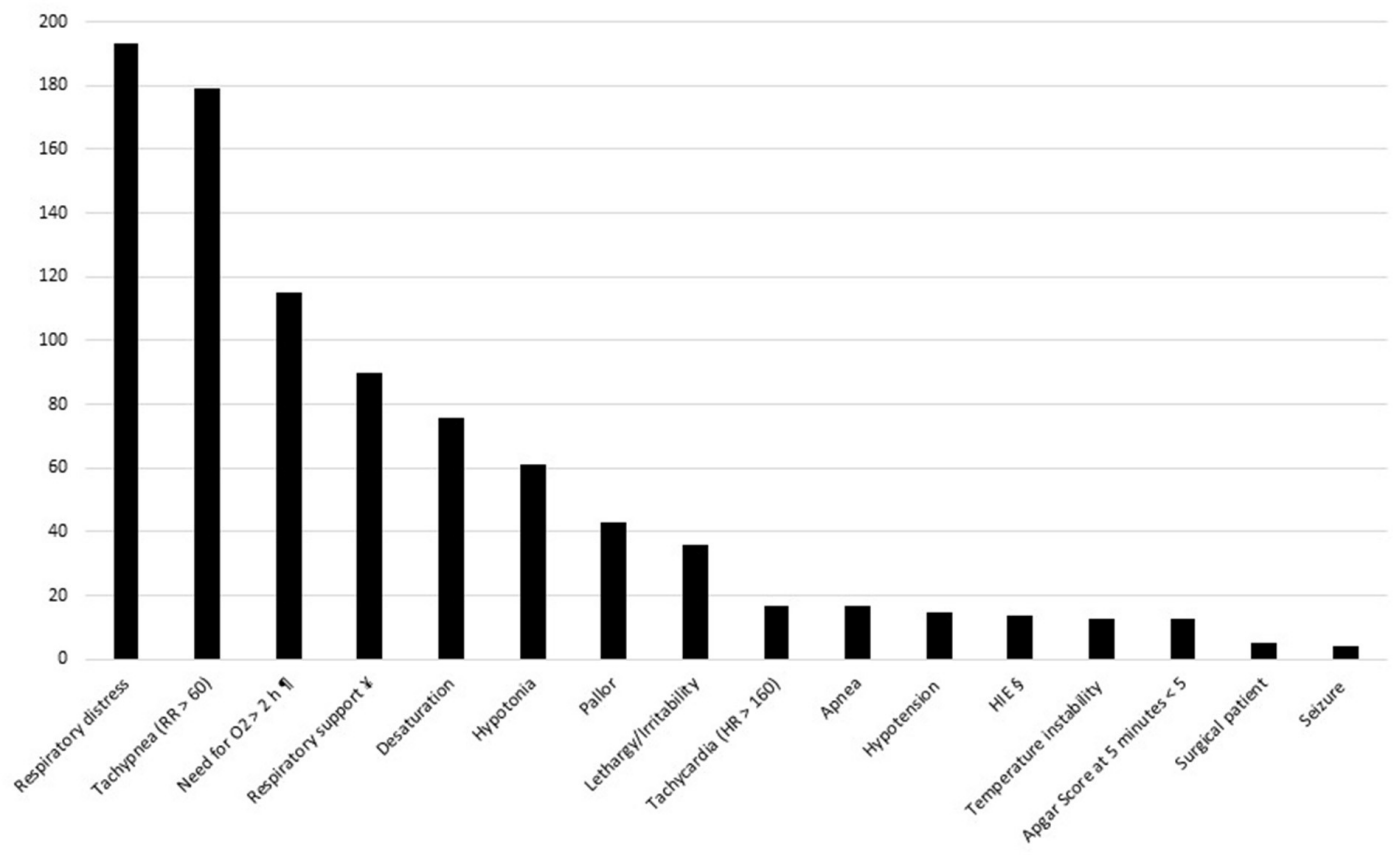
Symptoms and respiratory supports among symptomatic neonates. HIE, hypoxic ischemic encephalopathy. ¶ to maintain SpO2 > 90%. ¥ persistent need for NCPAP / HFNC / mechanical ventilation. § hypoxic ischemic encephalopathy requiring hypothermia.

Figure 1 details neonatal symptoms, Apgar scores and the need for respiratory support among symptomatic neonates. Respiratory symptoms (tachypnea, respiratory distress syndrome, desaturation) were the most common.

### Comparison between SCO and NSC

Among 3,445 infants, the following indications were suggested by NSC: no culture, no antibiotics, routine vitals (*n* = 3,238), strong consideration of starting empiric antibiotics, vitals per NICU (*n* = 131); empiric antibiotics, vitals per NICU (*n* = 55); no culture, no antibiotics, vitals every 4 h for 24 h (*n* = 13); and blood culture, vitals every 4 h for 24 h (*n* = 8).

Table 4 compares the number of “rule out sepsis” evaluations (a) and antibiotic treatments (b) in all neonates. Of the 3,380 neonates who did not start on antibiotics according to the SCO, 3,254 would also have avoided antibiotics according to the NSC. The remaining 126 neonates would have been recommended antibiotics by the NSC, but remained well without treatment. In contrast, five neonates would have avoided antibiotics according to NSC (absolute difference 3.5%; 95% CI, 3.1–3.7%, *p* <0.0001). According to NSC, antibiotics would have been administered to 5.4% of infants. Of the 138 neonates who were evaluated to rule out sepsis according to the SCO, 118 were also evaluated according to the NSC. Seventy-six neonates would have undergone “rule out sepsis” according to the NSC but were not evaluated. In contrast, 20 neonates would not have been evaluated according to as per NSC but were evaluated according to as per SCO (absolute difference, 1.63%; 95% CI, 1.10–2.05; *p* <0.0001). According to NSC, 5.6% of infants would have undergone “rule out sepsis.”

**Table 4:** Comparison of antibiotic use **(A)** and “rule out sepsis” evaluations **(B)** as per SCO vs. recommendations of the NSC in the study population. Differences between proportions were analyzed using McNemar's test.

		**NSC**		
		**No antibiotics**	**Antibiotics**	**Total (% of study cohort)**
**(A)** Comparison of antibiotic use				
**SCO**	No antibiotics	3,254	126	3,380 (98.1)
	Antibiotics	5	60	65 (1.9)
	Total (% of study cohort)	3,259 (94.6)	186 (5.4)	3,445 (100.0)
		**NSC**		
		**No test**	**Rule out sepsis**	**Total (% of study cohort)**
**(B)** Comparison of “rule out sepsis” evaluations				
**SCO**	Not evaluated	3,231	76	3,307 (96.0)
	Rule out sepsis	20	118	138 (4.0)
	Total (% of study cohort)	3,251 (94.4)	194 (5.6)	3,445 (100.0)

*NSC, neonatal sepsis calculator; SCO, serial clinical observation*.

Table 5 compares the number of “rule out sepsis” evaluations (a) and antibiotic treatments (b) among late-preterm infants. According to NSC, antibiotics would have been administered in 27.0% of late-preterm infants compared to 8.4% according to SCO (absolute difference, 18.6%; 95% CI, 12.3–24.8%; *p* <0.0001). Similarly, according to NSC, 28.1% of late-preterm infants would have undergone “rule out sepsis,” compared to 18.5% according to SCO (absolute difference, 9.6%; 95% CI, 4.4–14.7%; *p* <0.0001).

**Table 5:** Comparison of antibiotic use **(A)** and “rule out sepsis” evaluations **(B)** as per SCO vs. recommendations of the NSC among late-preterm infants.

		**NSC**		
		**No antibiotics**	**Antibiotics**	**Total (% of preterm infants)**
**(A)** Comparison of antibiotic use				
**SCO**	No antibiotics	130	33	163 (91.6%)
	Antibiotics	0	15	15 (8.4%)
	Total (% of preterm infants)	130 (73.0%)	48 (27.0%)	178 (100%)
		**NSC**		
		**No test**	**Rule out sepsis**	**Total (% of preterm infants)**
**(B)** Comparison of “rule out sepsis” evaluations				
**SCO**	Not evaluated	127	18	145 (81.5%)
	Rule out sepsis	1	32	33 (18.5%)
	Total (% of preterm infants)	128 (71.9%)	50 (28.1%)	178 (100%)

*NSC, neonatal sepsis calculator; SCO, serial clinical observation*.

*Differences between proportions were analyzed using McNemar's test*.

### Maternal temperature and neonatal symptoms

Among the 110 neonates born after an increased maternal intrapartum temperature (≥37.5°C), 20 developed symptoms. Eight of these 20 patients were treated with antibiotics. For the 12 untreated neonates, the NSC would have suggested the following: empiric antibiotics, vitals per NICU (*n* = 7); blood culture, vitals every 4 h for 24 h (*n* = 2); no culture, no antibiotics, routine vitals (*n* = 2); and strongly consider starting empiric antibiotics and vitals per NICU (*n* = 1). Therefore, 8 of the 12 neonates would have received antibiotics per NSC but remained untreated.

**Figure 2:**
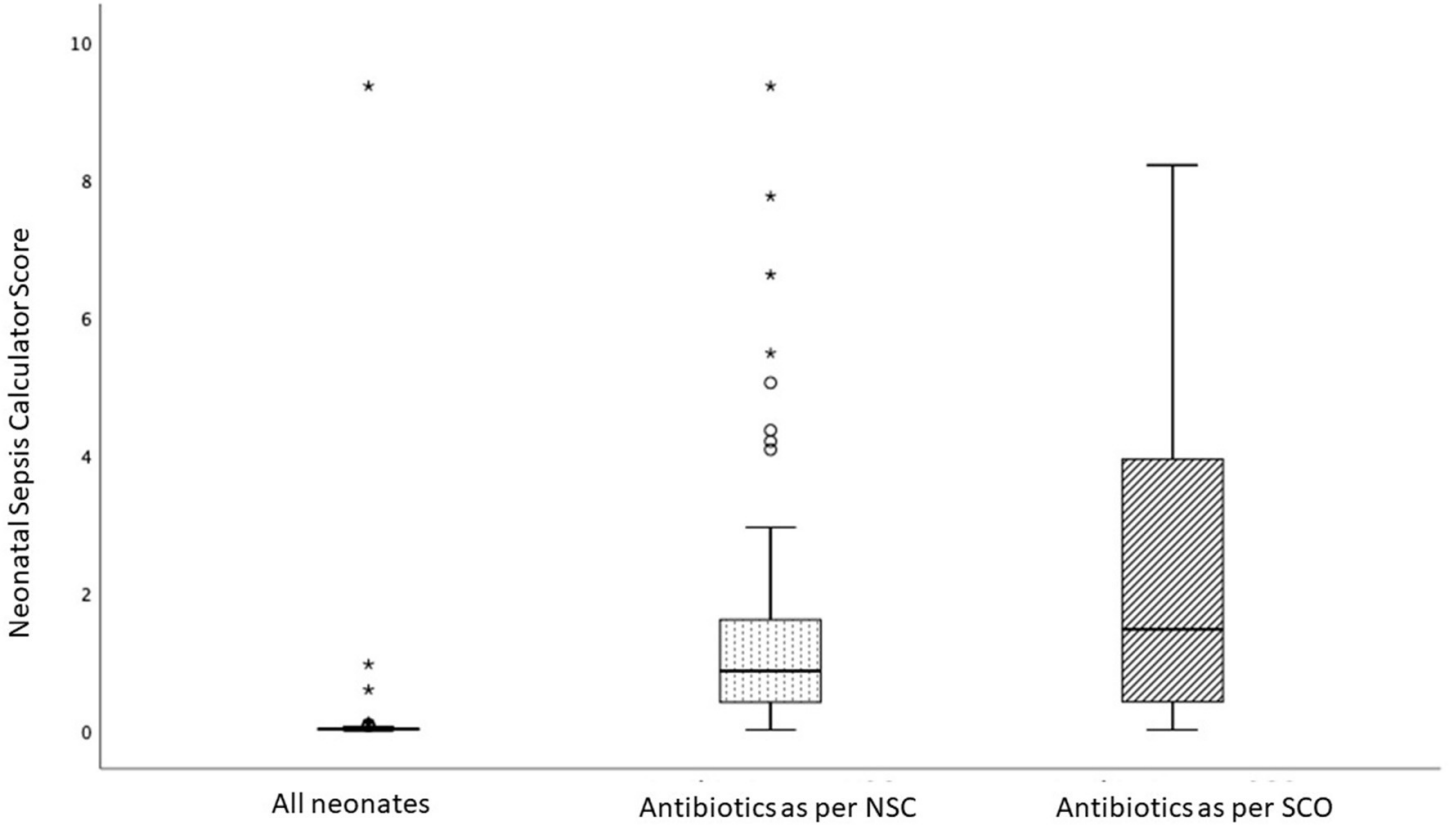
Box-and-Whisker plot comparing the score of the Neonatal Sepsis Calculator (NSC) in three groups: all infants in the study (median score = 0.02; IQR 0.02), infants receiving antibiotics as per NSC (median = 0.86; IQR = 1.22) and infants recommended antibiotics by Serial Clinical Observation (SCO) (median = 0.86; IQR = 1.22). The score in the study population was low; infants recommended antibiotics as per SCO had higher scores compared with infants recommended antibiotics as per NSC. Each box bounds the IQR range divided by the median (solid horizontal line); the lower and upper margins of the box represent the 25th and the 75th centile, respectively. The whiskers extend 1.5 times the IQR from the median. A circle (o) is used to mark outliers with values between 1.5 and 3 box lengths from the upper edge of the box; the asterisk (*) is used for extreme outliers (a value more than 3 times the interquartile range).

Figure 2 is a box plot showing the distribution of the NSC scores in the three cohorts: (i) all infants in the study population, (ii) infants initiated on antibiotics according to SCO, and (iii) those recommended antibiotics by NSC. The sepsis risk score in the study population was low; infants treated with antibiotics according to SCO had higher sepsis risk scores than those recommended antibiotics by the NSC.

### Neonatal outcome

No cases of culture-proven sepsis were found. Six infants were administered antibiotics (≥5 days), and all but one were born to GBS-negative mothers. Three of the six infants had an abnormal blood count, three had elevated CRP, and four underwent lumbar puncture (the CSF was sterile in all cases). Their blood cultures were sterile except for one neonate (born to a GBS-positive mother and exposed to inadequate IAP) who developed mild tachypnea (7 h of life) and received ampicillin plus gentamicin. The blood culture yielded *Staphylococcus hominis*, which was considered a contaminant because the subsequent blood culture was sterile.

None of the 3,445 included infants was readmitted within 30 days of birth with a positive blood culture result. Among neonates who developed symptoms after birth, none worsened or had brain lesions due to delayed antibiotic treatment. Two neonates died: the first was affected by an inherited metabolic disease and the second was affected by a congenital diaphragmatic hernia.

## Discussion

There is a consensus to administer empirical antibiotics to neonates with suspected EOS symptoms, with or without RFs (17). This strategy is based more on historical customs and practices than on evidence. In most babies, non-specific symptoms during the first hours of life are not due to infection. Unnecessary antibiotics may disrupt the neonatal gut microbiome with long-term consequences and increase the resistance to pathogens. Furthermore, intravenous infusion may complicate extravasation, whereas mother-infant separation for EOS evaluation can delay breastfeeding initiation and increase formula supplementation (7, 18). Therefore, strategies to reduce unnecessary antibiotic use are of interest. This is the first comparison between the NSC and the updated SCO approach (13). Indeed, we defined “minor” and “major” clinical symptoms to guide clinicians in the evaluation and treatment of infants with antibiotics.

Approximately ¾ of the symptomatic neonates were not exposed to antibiotics; 38% were allowed to “room in” with their mother within a few hours after birth. Among the remaining 62% who were admitted to the NICU or to the intermediate care unit, some underwent nCPAP or HFNC several hours after birth. Therefore, the persistence of symptoms beyond 2 h after birth may not be a good criterion for the administration of antibiotics, especially if the symptoms do not worsen or the risk of EOS is very low (i.e., infants born out of labor and with intact membranes). “Equivocal symptoms” or “clinical illness” are common in the first hours of life due to the transition to extrauterine life. The developers of NSC suggested that antibiotics “strongly consider” as a safeguard not to discontinue therapy in clinically symptomatic infants, even if the posterior probability is below the threshold for treatment (<3 cases/1,000 live births). However, a comparison with our updated SCO approach shows that this recommendation of NSC would substantially increase antibiotic exposure in uninfected infants without improving neonatal outcomes. In particular, our updated SCO approach reduced antibiotic exposure among late preterm infants by two-thirds compared to NSC. The NSC model is associated with increased postnatal antibiotic exposure, especially among infants with RFs for EOS or those with “transitional” symptoms in the first hours of life. Indeed, in some cases, the NSC model can overestimate the absolute risk of EOS; for example, the NSC assumes the same risk of EOS for neonates unexposed or exposed to inadequate IAP (duration <2 h) (19); however, it is known that the risk of developing EOS in asymptomatic IAP-exposed neonates is very low, regardless of the duration of IAP (20).

The low rate of “rule out sepsis” evaluations we performed in infants with mild symptoms probably contributed to the reduction of unnecessary antibiotics. This finding is consistent with recent studies demonstrating the low predictive value of ancillary tests (21) and the reduced (~30%) antibiotic exposure when CRP is excluded from the diagnostic panel of EOS (22). Furthermore, the importance of a positive blood culture obtained from an asymptomatic newborn infant is unclear (23). In our experience, repeated evaluations of asymptomatic infants may even increase the yield of pathogens from blood cultures (often difficult to interpret), thus giving antibiotics even to infants whose symptoms of EOS would likely never appear.

Only 1.9% of our infants were exposed to antibiotics and most received very short courses. Our approach was of utmost benefit in preterm infants, who often have “transient” symptoms in the first few hours of life, compared with full-term infants. Until recently, up to 35% of late-preterm infants received antibiotics (24), thus separating neonates from their mothers. In contrast, only 8% of our preterm neonates received antibiotics, while 70% were allowed to room in with their mothers. However, the overall neonatal antibiotic exposure rates in preterm and full-term neonates could be 30 times higher than necessary, as the incidence of EOS in our NICU is 0.6/1,000 live births (16). More efforts should be made to better identify the infants to be treated. None of the neonates had worse outcomes due to delayed treatment of an infection. Perhaps the SCO strategy is safer in our center, as adherence to recommendations for GBS prevention is very high (25), whereas SCO would be less effective in centers with low adherence to guidelines.

This study had several important limitations. First, the SCO strategy may be safer in our center, where adherence to recommendations for the prevention of GBS is very high, while it could be less effective where adherence to guidelines is low, or the incidence of EOS is higher. Second, the sample size of the infants in the study was small and we had no cases of culture-proven EOS to define a hypothetical overtreatment index or to confirm the safe management of infected neonates, although this information has already been provided in our previous study (10). In addition, management was based on SCO, whereas the data elements for the NSC were collected retrospectively. This would result in a less accurate identification of the symptoms by which the NSC score was calculated, although newborn charts accurately describe their clinical condition. Finally, the definition of “minor” and “major” symptoms was defined a priori, based on expert opinions in our network. However, a large prospective study including EOS cases and controls may more accurately define which symptoms are most predictive of EOS.

In conclusion, SCO of asymptomatic infants with RFs for EOS or with “mild, non-progressive symptoms” during the first days of life reduces laboratory evaluation, minimizes unnecessary antibiotics, and avoids separation of the mother from her infant without delaying antibiotic treatment of infected infants. Antibiotic overuse is a planetary emergency and we hope that our experience can help reduce neonatal antibiotic exposure.

## Data availability statement

The original contributions presented in the study are included in the article/supplementary material, further inquiries can be directed to the corresponding author.

## Ethics statement

The studies involving human participants were reviewed and approved by Comitato Etico Area Vasta Emilia Nord. Written informed consent from the participants' legal guardian/next of kin was not required to participate in this study in accordance with the national legislation and the institutional requirements.

## Author contributions

AB conceptualized the study, drafted the initial manuscript, reviewed and edited it, and supervised the study. IZ and LL contributed to conceptualization, drafted the initial reviewed, and edited the manuscript. LB, EV, AT, GT, ML, FL, FMo, MC, TZ, AB, LI, and FMi contributed to acquisition, analysis and interpretation of data for the work, and reviewed and edited the manuscript. All authors substantially contributed to the work, gave final approval of the version to be published, and agree to be accountable for all aspects of the work.

## Funding

This study was partially supported by the Agenzia Sanitaria Regionale (Emilia-Romagna) Piano Regionale della Prevenzione (2015-2018) C.U.P. n. E43G17000680002.

## Conflict of interest

The authors declare that the research was conducted in the absence of any commercial or financial relationships that could be construed as a potential conflict of interest. The handling editor CA declared a past co-authorship with one of the authors AB.

## Publisher's note

All claims expressed in this article are solely those of the authors and do not necessarily represent those of their affiliated organizations, or those of the publisher, the editors and the reviewers. Any product that may be evaluated in this article, or claim that may be made by its manufacturer, is not guaranteed or endorsed by the publisher.
